# Supplementary material for: Septins Enable T Cell Contact Guidance via Amoeboid-Mesenchymal Switch
Source: bioRxiv. 2023 Sep 27:2023.09.26.559597. Preprint. [Version 1] doi: 10.1101/2023.09.26.559597 (PMC10557721; doi:10.1101/2023.09.26.559597)
Supplement: Supplement 4 [file NIHPP2023.09.26.559597v1-supplement-4.pdf]

bioRxiv preprint doi: https://doi.org/10.1101/2023.09.26.559597; this version posted September 27, 2023. The copyright holder for this preprint (which was not certified by peer review) is the author/funder, who has granted bioRxiv a license to display the preprint in perpetuity. It is made available under aCC-BY 4.0 International license.

MOVIE LEGENDS

Movie 1. Wide-field view of hCD4+ T cell migration on 2D (flat) ICAM1 parallel lanes micropattern during their transitioning from predominantly amoeboid 'random walk' towards a mesenchymal-like contact guidance mode after the addition of UR214-9 septin inhibitor.

Movie 2. Detailed zoom-in view of hCD4+ T cell mesenchymal-like contact guidance along ICAM1 micro-lanes.

Movie 3. Wide-field view of the hCD4+ T cells' contact guidance along ICAM1 micro-lanes during prolonged **+UR214-9** treatment (t > 1 hour).

Movie 4. hCD4+ T cells predominantly amoeboid 'random walk' meandering on the flat 2D ICAM1 grids.

Movie 5. UR214-9-induced hCD4+ T cells mesenchymal-like spreading and formation of dendritic-like cell protrusions along the ICAM1 grid.

Movie 6. Non-responsiveness of UR214-9-induced mesenchymal-like hCD4+ T cell dendritic adhesion, spreading, and contact guidance dynamics to Blebbistatin-mediated suppression of actomyosin contractility.

Movie 7. Dynein contractility inhibition with Dynapyrazole A abrogates UR214-9-induced hCD4+ T cells mesenchymal-like adhesion, spreading, and migration along ICAM1 grids. *Note that the tested T cells are co-treated with UR214-9 and Blebbistatin to demonstrate dispensable status of actomyosin contractility during mesenchymal-like T cell contact guidance mode under UR214-9 treatment.*

Movie 8. Abrogation of hCD4+ T cell's UR214-9-induced mesenchymal-like adhesion, spreading, and migration with Dynapyrazole A-induced dynein inhibition (no Blebbistatin co-treatment). Following washout demonstrates partial reversibility of treatment effects.

Movie 9. Inhibition of the actomyosin contractility **(+Blebbistatin)**, unlike UR214-9 treatment, does not induce mesenchymal-like ‘dendritic’ cell spreading.

Movie 10. Sole inhibition of the dynein contractility **(+Dynapyrazole A)** does not change hCD4+ T cell predominantly amoeboid migratory behavior on ICAM1 grids.

bioRxiv preprint doi: https://doi.org/10.1101/2023.09.26.559597; this version posted September 27, 2023. The copyright holder for this preprint (which was not certified by peer review) is the author/funder, who has granted bioRxiv a license to display the preprint in perpetuity. It is made available under aCC-BY 4.0 International license.

SUPPLEMENTARY FIGURES

a UR214-9-to-Septin docking *in silico* modeling, as compared to GTP, GDP and FCF :

(**1**) Dockings views for pocket 1 — (**2**) Dockings views for pocket 2

Septin-2, Septin-6, Septin-7, Septin-9

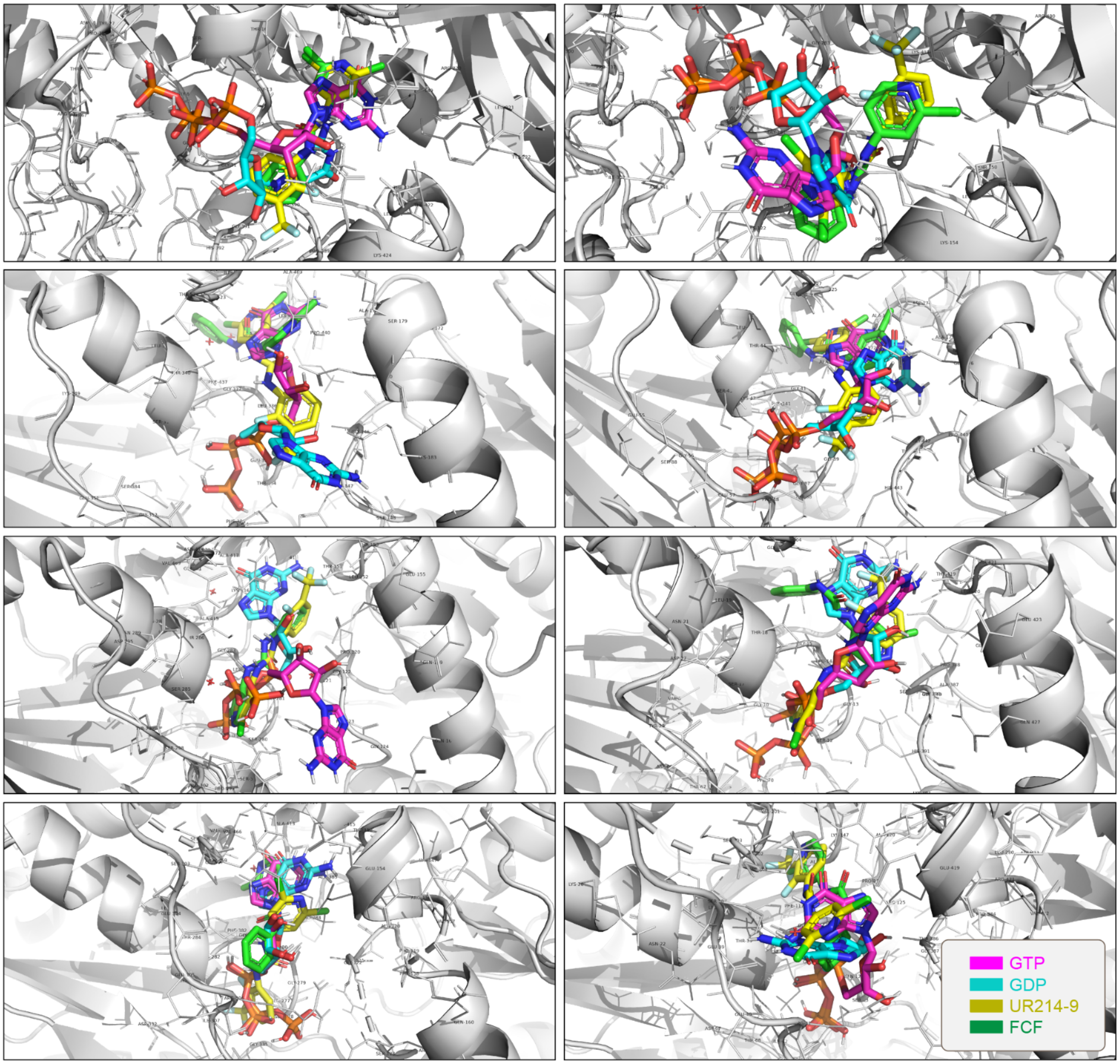

b UR214-9-to-Septin docking energy *in silico* modeling, as compared to GTP, GDP and FCF :

(**1**) Table 1

Ligand	Energy of interactions, pocket 1 (kcal/mol):			
	Septin-2	Septin-6	Septin-7	Septin-9
GTP	-41.4013	-42.7594	-37.1021	-42.9431
GDP	-36.3165	-35.7889	-34.5495	-37.3714
UR214-9	-33.4929	-32.3030	-32.5959	-34.9654
FCF	-27.4643	-31.9061	-26.8665	-27.6648

(**2**) Table 2

Ligand	Energy of interactions, pocket 2 (kcal/mol):			
	Septin-2	Septin-6	Septin-7	Septin-9
GTP	-41.5791	-42.7328	-39.3803	-42.1438
GDP	-36.7661	-37.8403	-38.6942	-37.0604
UR214-9	-32.6248	-32.2466	-32.2272	-33.0542
FCF	-26.6455	-31.5510	-27.6554	-26.6600

bioRxiv preprint doi: https://doi.org/10.1101/2023.09.26.559597; this version posted September 27, 2023. The copyright holder for this preprint (which was not certified by peer review) is the author/funder, who has granted bioRxiv a license to display the preprint in perpetuity. It is made available under aCC-BY 4.0 International license.

Supplemental Figure SI1. UR214-9-septin *in silico* docking simulations display competitive energies of interactions as compared to FCF, GTP and GDP.

(a) *In silico* computational model for UR214-9 docking to septin-2, -6, -7 and -9's GTPase site at the respective pockets 1 and 2 in septin molecules, as compared to FCF inhibitor and GTP.

(b) *In silico*-calculated docking energies for GTP, GDP, UR214-9 and FCF.

bioRxiv preprint doi: https://doi.org/10.1101/2023.09.26.559597; this version posted September 27, 2023. The copyright holder for this preprint (which was not certified by peer review) is the author/funder, who has granted bioRxiv a license to display the preprint in perpetuity. It is made available under aCC-BY 4.0 International license.

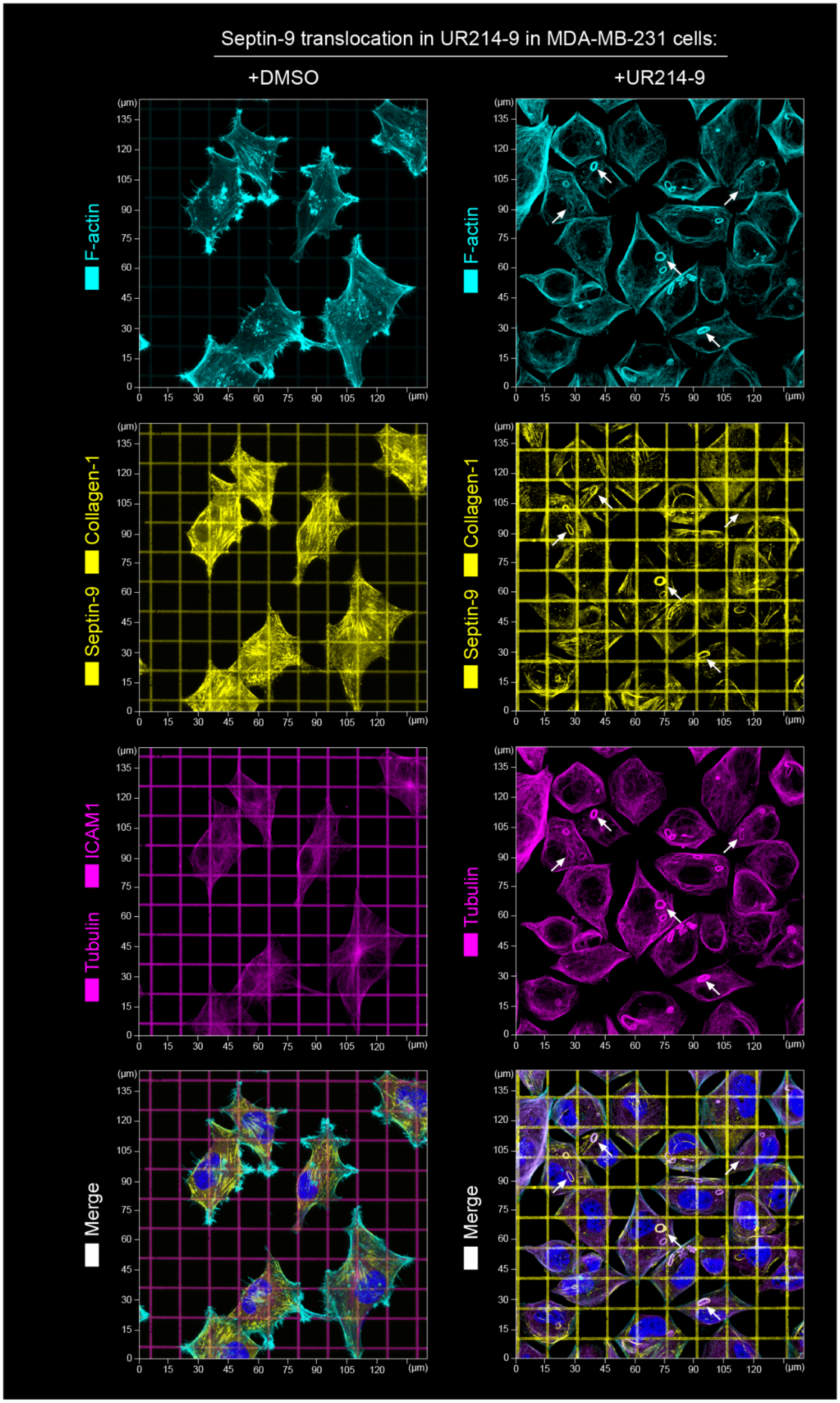

bioRxiv preprint doi: https://doi.org/10.1101/2023.09.26.559597; this version posted September 27, 2023. The copyright holder for this preprint (which was not certified by peer review) is the author/funder, who has granted bioRxiv a license to display the preprint in perpetuity. It is made available under aCC-BY 4.0 International license.

Supplemental Figure SI2. Septins inhibition (+UR214-9) in MDA-MB-231 cells on collagen (type I) grids results in translocation of septin-9 from the stress-fibers onto microtubules.

Left - Septin-9 in vehicle-treated cells **(+DMSO)** colocalizes with linear F-actin structures (stress-fibers).

Right - Septin-9 translocates onto the microtubules upon suppression of septins GTPase activity **(+UR214-9,** t≥1 hour**).**

Note that inactivation of GTPase activity if septins leads to the increased density of the MT network.

Note that GTPase-inactive septin-9 acts as a microtubule-associated protein (MAP), stabilizing microtubules and inducing MT curling into the rings (arrows) (37).

bioRxiv preprint doi: https://doi.org/10.1101/2023.09.26.559597; this version posted September 27, 2023. The copyright holder for this preprint (which was not certified by peer review) is the author/funder, who has granted bioRxiv a license to display the preprint in perpetuity. It is made available under aCC-BY 4.0 International license.

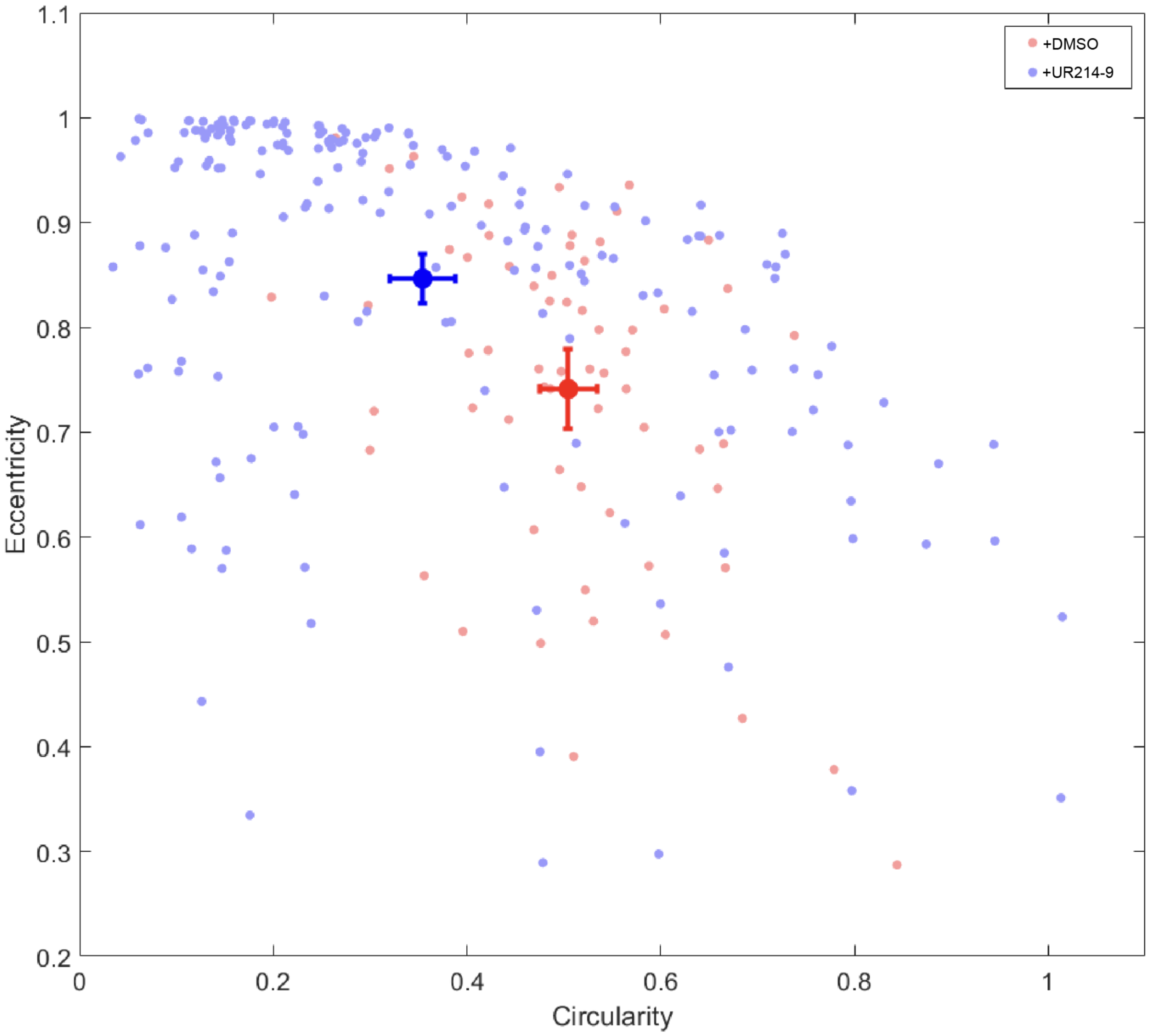

Supplemental Figure SI3. Analysis of hCD4+ T cells morphological response to the inhibition of the septins with UR214-9.

A cross-correlated substantial eccentricity (T cell elongation) and circularity ($4\pi S/L^2$, where S is the projected cell area, L is projected cell perimeter) changes. *Note the increase in cell elongation (eccentricity) and decrease of T cell circularity upon treatment with UR214-9.*

bioRxiv preprint doi: https://doi.org/10.1101/2023.09.26.559597; this version posted September 27, 2023. The copyright holder for this preprint (which was not certified by peer review) is the author/funder, who has granted bioRxiv a license to display the preprint in perpetuity. It is made available under aCC-BY 4.0 International license.

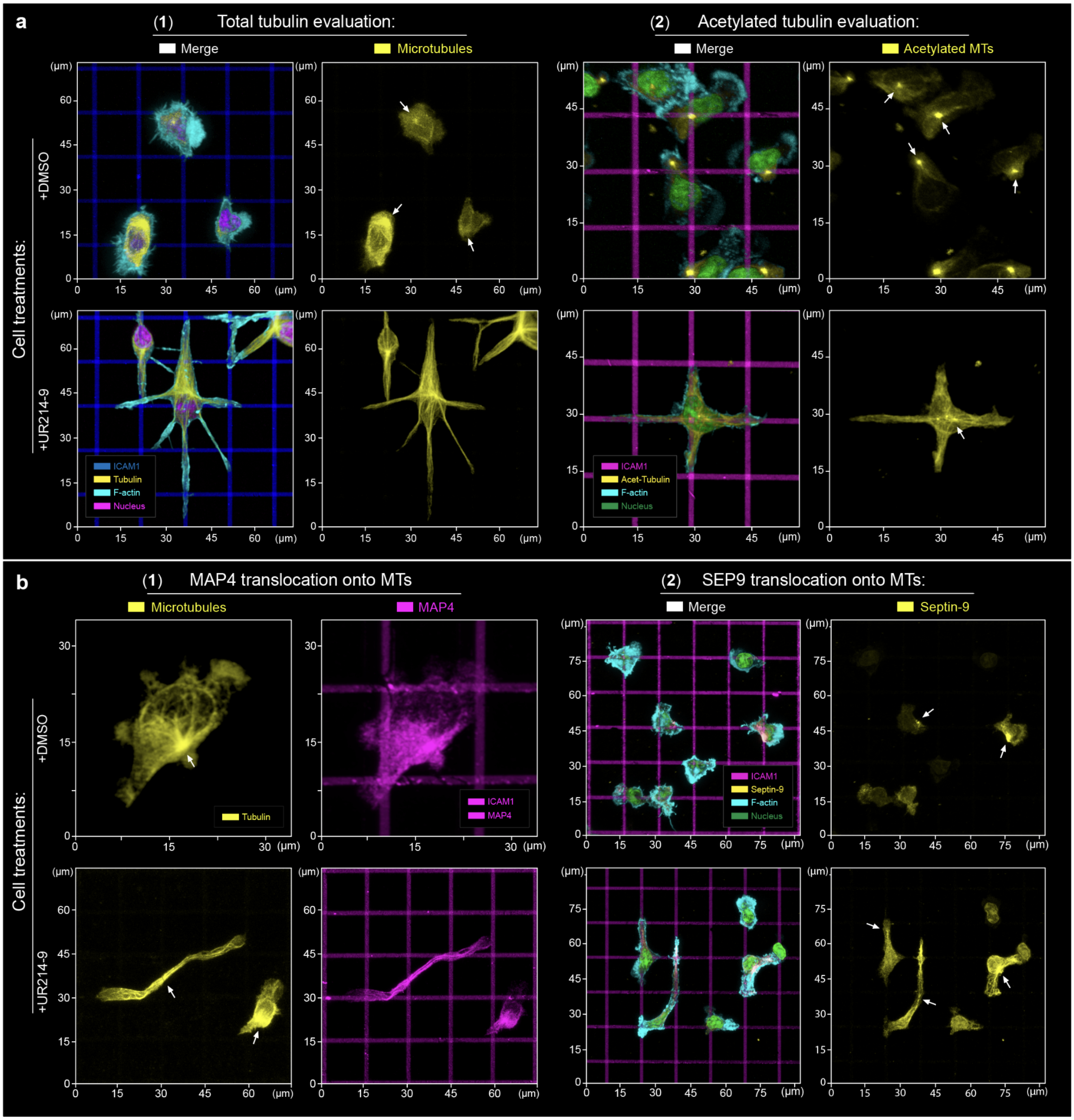

Supplemental Figure SI4. Immunofluorescence analysis of the post-translational microtubule modifications in UR214-9-treated cells.

(a) Both microtubules density (**1**), and MT acetylation (**2**) are substantially enhanced upon UR214-9-induced suppression of septin GTPase activity **(+UR214-9)**, compared to the diffuse cytoplasmic tubulin and sparse and short microtubules in the control T cells **(+DMSO)**. **(b)** Both MAP4 (**1**) and SEP9 (**2**) translocate to the microtubules upon inhibition of septins with UR214-9.

bioRxiv preprint doi: https://doi.org/10.1101/2023.09.26.559597; this version posted September 27, 2023. The copyright holder for this preprint (which was not certified by peer review) is the author/funder, who has granted bioRxiv a license to display the preprint in perpetuity. It is made available under aCC-BY 4.0 International license.

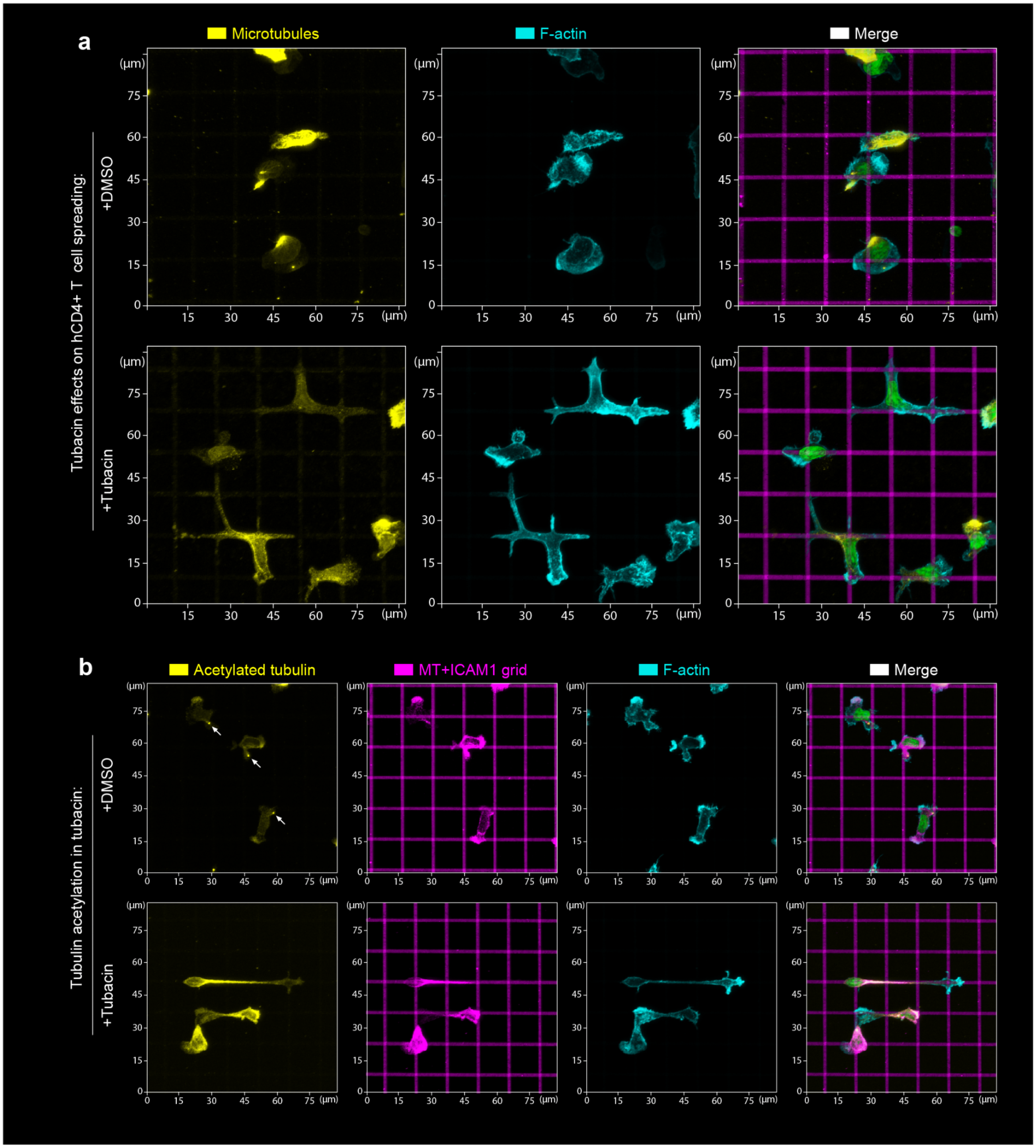

Supplemental Figure SI5. Stabilization of microtubules *via* Tubacin-induced hyperacetylation replicates (*i.e.*, phenocopies) the UR214-9-induced hCD4+ T cell transitioning towards mesenchymal-like dendritic spreading.

(a) hCD4+ T cells during HDAC6 suppression **(+Tubacin,** t≥1 hour**)** display a partial amoeboid-to-mesenchymal-like transition, *i.e.* T cell dendritic spreading along the ICAM1 grids.

(b) Analysis of MT acetylation in hCD4+ T cells, induced by HDAC6 suppression **(+Tubacin,** t≥1 hour**),** shows an increased acetylation of microtubules compared to the control T cell group **(+DMSO)**. *Identified centrosomes indicated with arrows.*
